# Comparison of the Effectiveness of Intra-articular Infiltration of Hyaluronic Acid and Corticosteroids in the Management of Knee Osteoarthritis: A Prospective Comparative Study

**DOI:** 10.7759/cureus.50449

**Published:** 2023-12-13

**Authors:** Tayyab Mumtaz Khan, Muhammad Zeshan Mehmood, Javaria Riaz, Zoya Nawaz, Hamid Arshad, Haider Ali, Muhammad Hamza Riaz, Osama Afzal, Asim Ali, Muhammad Hassan Ahmad, Rana Shahzaib Ali

**Affiliations:** 1 Orthopaedic Surgery, Rawalpindi Medical University, Rawalpindi, PAK; 2 Surgery, Benazir Bhutto Hospital, Rawalpindi, PAK; 3 Medicine, Mohi-ud-Din Islamic Medical College, Mirpur, PAK; 4 Pathology and Laboratory Medicine, Lahore General Hospital, Lahore, PAK; 5 Surgery, Allama Iqbal Medical College, Lahore, PAK; 6 Medicine, Allama Iqbal Medical College, Lahore, PAK; 7 Coronary Care Unit, Allama Iqbal Medical College, Lahore, PAK; 8 Cardiology, Pakistan Kidney & Liver Institute and Research Centre, Lahore, PAK; 9 Surgery, Rawalpindi Medical University, Rawalpindi, PAK; 10 Orthopaedic Surgery, Sheikh Zayed Medical College and Hospital, Rahim Yar Khan, PAK

**Keywords:** comparative study, prospective, knee osteoarthritis, management, corticosteroids, hyaluronic acid, intra-articular, effectiveness, comparison

## Abstract

Background

Knee osteoarthritis (KOA) is a chronic and progressive disease of the knee joint characterized by articular cartilage destruction. It is the most common cause of knee disability and pain globally. Various treatments are used for the management of KOA; however, the role of intra-articular injections in KOA management in Pakistan remains understudied. Therefore, this study aims to evaluate the effectiveness of intra-articular injections of hyaluronic acid (HA) and corticosteroids in the management of KOA.

Methodology

This randomized, prospective, comparative study was conducted among 88 patients diagnosed with KOA in the outpatient department clinic of orthopedics in Benazir Bhutto Hospital, Rawalpindi, from January 2022 to January 2023. For patient enrolment, structured inclusion and exclusion criteria and a simple random sampling technique were used. Before data collection, ethical approval and informed consent were obtained. Data collection was done via a self-structured and interview-based proforma. Data analysis was performed through descriptive statistics and independent t-tests using SPSS version 25 (IBM Corp., Armonk, NY, USA).

Results

KOA was more prevalent in women (60, 68.18%) than men (28, 31.82%). The means for study variables such as age, Visual Analog Scale (VAS) score, and Western Ontario and McMaster Universities (WOMAC) score were 58.08 ± 7.89 years, 7.66 ± 1.8, and 71.86 ± 8.90, respectively. The incidences of right-sided and left-sided KOA were 57 (64.77%) and 31 (35.23%), respectively. Likewise, the frequency of grade II KOA was 55 (62.50%), while the frequency of grade III KOA was 33 (37.50%). Differences in the mean scores of both VAS and WOMAC between study groups were statistically significant at the second-week, sixth-week, and third-month follow-up visits. However, the mean scores of VAS and WOMAC were lower in group B than in group A at the second-week follow-up visit, whereas the scores were lower in group A compared to group B after the sixth week and third month of intra-articular injections.

Conclusions

Intra-articular injections of both HA and corticosteroids were adequately effective in the management of KOA-associated pain and functional restrictions; nevertheless, the benefits of corticosteroids were acute and short-term, whereas the outcomes of HA were gradual and long-term.

## Introduction

Knee osteoarthritis (KOA) is a progressive and degenerative disease of the knee joint which is characterized by the deterioration of the articular cartilage, osteophyte formation, and narrowing of the joint space. Its symptoms include joint pain, swelling, stiffness, and restriction in joint movement [1,2]. The global prevalence of the KOA is 18% among women and 9.6% among men [3]. The prevalence of KOA in Pakistan is also high as a study reported that 28% of the urban and 25% of the rural Pakistani population suffer from KOA [4].

KOA is one of the major causes that lead to knee discomfort and restriction in movement. It is estimated that KOA impacts movement in 80% of patients, with 25% of patients unable to even perform their routine activities [3]. Difficulty in daily activities results in worsening social and financial aspects of life in the affected population. KOA not only impacts the physical health of patients but also compromises the mental health of patients. It is well known that patients with KOA have a high incidence of anxiety and depression [5]. Furthermore, it also consumes a large amount of health resources. In the United States, osteoarthritis burden costs almost 128 billion USD annually [6].

Development of KOA is associated with many factors such as old age, female gender, family history, obesity, heavy weight lifting, knee injury, sedentary lifestyle, and abnormal body positions [1-5]. The changes in KOA at the molecular level include chondrocyte senescence. Chondrosenescence refers to the age-linked decrease in chondrocyte functions. Furthermore, cytokines and many other growth factors also contribute to the proteolytic deterioration of joint cartilage which leads to KOA [7].

Treatment modalities of KOA begin with non-pharmacological interventions (weight loss, diet modification, physiotherapy, and brace), followed by systemic analgesic drugs (non-steroidal anti-inflammatory drugs and opioids), local treatment strategies (topical analgesics and intra-articular injections), and ends with surgery as the last option [7,8].

Intra-articular administration of hyaluronic acid (HA) restores knee function and relieves pain by fulfilling the deficiency of HA which is a main component of joint cartilage and synovial fluid and works as a lubricator and shock absorber in joints. HA gets depleted during osteoarthritis development [9]. Intra-articular injections of corticosteroids leads to improvement in KOA patients via a reduction in inflammatory changes in the joints by suppressing T and B-cell functions and other inflammatory mechanisms [10].

In the literature, several global studies have reported the effectiveness of intra-articular injections in the management of KOA; however, studies comparing the effectiveness of intra-articular injections of HA and corticosteroids are lacking, especially from Pakistan. Therefore, this study aimed to assess the effectiveness of intra-articular administration of HA and corticosteroids in the management of KOA.

## Materials and methods

Study design and population

This randomized, prospective, comparative study was conducted in the outpatient department of orthopedics in Benazir Bhutto Hospital, Rawalpindi, among 88 patients diagnosed with unilateral KOA from January 2022 to January 2023. The sample size was calculated by the World Health Organization calculator. Using simple random sampling, patients were divided into equal groups, i.e., group A comprised 44 patients who were given an intra-articular injection of HA while group B comprised 44 patients who were given an intra-articular injection of corticosteroids.

Inclusion and exclusion criteria

Inclusion and exclusion criteria were applied to enroll participants in the study. Patients aged fifty or above of any gender, those with knee joint pain for at least three months, those with radiographically confirmed II or III grade of KOA (according to Kellgren Lawrence system of classification of osteoarthritis), those with conservative treatment failure for KOA after three months of its start, and those who showed willingness for participation and had attended follow up visits at the second week, sixth week, and third month after intra-articular injections treatment were included in the study. Patients who had a history of knee trauma/surgery, knee deformity, autoimmune disease, rheumatoid arthritis, bleeding disorder, and intra-articular injections in the last three months in the affected knee joints and those who refused participation and follow-up visits were excluded from the study.

Ethical considerations

Before the start of the study, ethical approval was obtained from the Ethical Review Board (ERB) of Benazir Bhutto Hospital, Rawalpindi (approval number: BBH.ERB.283/189). Informed consent from all participants was obtained after explaining the aims of the study.

Intra-articular administration of hyaluronic acid and corticosteroids

Intra-articular injections of HA (6 mL/48 mg of sodium hyaluronate) and corticosteroids (1 mL/40 mg of triamcinolone acetonide) were administered to patients’ knee joint synovial space via anterolateral approach after confirmation of synovial space by aspirating synovial fluid, with the affected knee flexed at 90 degrees. In both types of injections, 1 mL of 2% lignocaine was also mixed. Furthermore, strict aseptic measures and the no-touch method of the needle were followed for the administration of both types of injections. After intra-articular injections, aseptic dressing was done, and knee caps were advised to all patients.

Data collection

A self-structured proforma was used for data collection. It was composed of two components. Demographic data of the study population such as age and gender (male or female) was recorded in the first component of the proforma. Clinical assessment of the patients at their baseline, second-week, sixth-week, and third-month visits was noted on the second component of the proforma. Clinical evaluation of the patients regarding pain and function of the affected knee with osteoarthritis was made using the Visual Analog Scale (VAS) and Western Ontario and McMaster Universities (WOMAC) index. VAS measures pain intensity and its value ranges from 0 (no pain) to 10 (worst possible pain). WOMAC has three subscales related to pain, joint stiffness, and function, with scores ranging from 0 to 96. Lower scores imply less pain, less joint stiffness, and less limitation in joint function, while higher scores imply more pain, more joint stiffness, and more limitation in joint function. VAS and WOMAC have been used in international studies as well [10]. Data regarding the side of the affected knee and the grade of KOA were also noted on the second component of the proforma.

Data analysis

Data analysis was done via descriptive and inferential statistics using SPSS version 25 (IBM Corp., Armonk, NY, USA). Frequency and percentage of qualitative data were measured while means with standard deviation (SD) of the quantitative data were calculated using descriptive statistics. Independent-sample t-test was applied to compare the means of VAS and WOMAC scores between the two groups at different time intervals. P-values less than 0.05 were considered statistically significant.

## Results

Of the 88 patients, 60 (68.18%) were women, and 28 (31.82%) were men. The mean age, VAS score, and WOMAC score were 58.08 ± 7.89 years, 7.66 ± 1.8, and 71.86 ± 8.90, respectively. In 57 (64.77%) patients, the right knee was affected by osteoarthritis, whereas in the remaining 31 (35.23%) patients, the left knee was affected. Likewise, regarding the grade of KOA, 55 (62.50%) patients had grade II KOA, and 33 (37.50%) patients had grade III KOA.

Table 1 indicates that the difference in the means of VAS scores between group A and group B was insignificant at the start of the study, while the difference in the means of VAS scores between the study groups was significant at the second-week, sixth-week, and third-month follow-up visits. In group A, the mean VAS score was in a declining trend till the sixth week; however, after the third month of treatment, the VAS score started to rise gradually. In group B, this declining trend of the VAS mean score was faster; nevertheless, it was of short duration as the mean VAS score started to increase at the sixth-week follow-up visit.

**Table 1:** Comparison of mean VAS scores at various time intervals in the study groups and the t-test analysis. VAS = Visual Analog Scale

Parameters	VAS scores		Independent t-test
Time intervals	Group A	Group B	P-value
Baseline	7.59 ± 1.70	7.74 ± 1.10	0.50
Second week	3.60 ± 0.90	2.55 ± 1.44	0.04
Sixth week	2.87 ± 1.03	3.60 ± 1.18	0.02
Third month	3.10 ± 1.33	4.80 ± 1.20	0.01

Table 2 shows that the variation in the mean WOMAC scores between group A and group B was insignificant at the initial visit; however, the difference in the mean WOMAC scores between the study groups was significant in the second week, sixth week, and third month after intra-articular injection administration. In group A, the mean WOMAC score was in a declining trend until the sixth-week follow-up visit; nevertheless, at the third-month follow-up visit, the mean WOMAC score began to rise gradually. In group B, the downward shift of the mean WOMAC score was swift, although it was for a brief period as the mean WOMAC score started to rise at the sixth-week post-treatment visit.

**Table 2:** Comparison of mean WOMAC scores at different periods in the study groups and the t-test analysis. WOMAC = Western Ontario and McMaster Universities

Parameters	WOMAC scores		Independent t-test
Time intervals	Group A	Group B	P-value
Baseline	70.56 ± 5.80	71.30 ± 3.06	0.80
Second week	60.23 ± 4.09	55.80 ± 8.08	0.01
Sixth week	55.68 ± 6.70	58.47 ± 8.01	0.04
Third month	57.46 ± 7.10	63.08 ± 8.76	0.02

## Discussion

This study compared the effectiveness of intra-articular injections of HA and corticosteroids in patients with KOA at different time intervals. Furthermore, it highlighted the difference in the frequency of KOA based on gender, affected side of the joint, and grade of KOA.

In this study, KOA was more common among women than men. Similar results regarding a higher prevalence of KOA among females, especially after menopause, than males have been reported in various global studies. A higher incidence of osteoarthritis after menopause among women can be due to low estrogen as it plays an anti-inflammatory role [2-4]. In this study, the right-sided knee joint was more affected by KOA than the left-sided knee joint. This asymmetry in the distribution of KOA can be constitutional, physiological, and pathological. Moreover, in most people, the right side of the body is the dominant side. Therefore, the right side of the knee joint is used more than the left and faces more repetitive stress than the left, leading to a higher incidence of KOA on the right side. A previous study supported this finding by noting a higher incidence of KOA on the right side [11].

After administration of intra-articular HA and corticosteroids into the synovial space of knee joints of patients, first, the mean VAS scores were compared between the two groups at various time intervals. For group A, VAS scores at different periods indicated that HA had a gradual and long-lasting impact on the management of KOA. For group B, the mean VAS scores at various patient visits suggested that corticosteroids provided acute and short-term improvement in patients with KOA. The difference in the mean VAS scores at various time intervals between the study groups was significant statistically, except during the initial visit of the patients.

The mean WOMAC scores were also compared between the two groups at various time intervals after administering intra-articular injections. For group A, the mean scores manifested the slow and long-term effects of HA in patients with KOA. For group B, the mean WOMAC scores for corticosteroids highlighted that corticosteroids provide fast but brief relief to patients with KOA. The difference in the WOMAC score means at different periods between the study groups was significant statistically, except at the baseline visit of patients.

In the literature, several studies have reported results similar to this study. A study from the United States suggested that HA provides gradual and long-lasting improvement to patients with KOA; on the other hand, corticosteroids provide swift but short-lasting improvement [12]. Another study from China reported consistent findings regarding the role of HA and corticosteroids in the management of KOA [13]. Likewise, an Iranian study also endorsed that both HA and corticosteroids are effective in the management of KOA; however, the effects of HA last for a longer duration, while the effects of corticosteroids last for a shorter duration [14]. A meta-analysis also supported the findings of this study [15]. The effects of corticosteroids in the management of KOA are acute and short-lived because they alter the inflammatory mechanism for a short duration, while HA benefits are gradual and long-lasting as it plays a role in the reconstruction of the articular cartilage and synovial fluid of joints which last for a longer duration [7-10]. Hence, we recommend corticosteroids for acute management of KOA while HA for long-term management of KOA.

This study has some limitations such as a single-centered study, small sample size, and follow-up at short intervals. Because of these limitations, the findings of this study can only be applied to small or regional populations. To generalize these results regarding the intra-articular injections of HA and corticosteroids in the management of KOA, multicenter studies with a large sample size and longer follow-ups are required.

## Conclusions

This study has demonstrated that intra-articular injections of HA and corticosteroids are adequately effective in the management of KOA-associated pain and functional limitations. Corticosteroid injections provide swift relief in both pain and functional restrictions; nevertheless, for a shorter duration. On the other hand, HA brings gradual and long-lasting improvement in patients with KOA. Furthermore, the differences in the mean VAS and WOMAC scores were significant statistically at all three follow-up visits between the study groups. The mean VAS and WOMAC scores were lower in group B compared to group A at the second-week post-treatment visit, while the scores were lower in group A compared to group B at the sixth-week and third-month follow-up visits. Therefore, this study recommends corticosteroids for the acute management of KOA and hyaluronic acid for the long-term management of KOA.
